# Starving infecund widow spiders maintain sexual attractiveness and trade off safety for enhanced prey capture

**DOI:** 10.1016/j.isci.2024.110722

**Published:** 2024-08-13

**Authors:** Andreas Fischer, Natalie De Vita, Sophia Phillips Sproule, Gerhard Gries

**Affiliations:** 1 Department of Biological Sciences, Simon Fraser University, 8888 University Drive, Burnaby, British Columbia V5A 1S6, Canada; 2 Department of General and Systematic Zoology, University of Greifswald, Loitzer Str. 26, 17489 Greifswald, Germany

**Keywords:** Biological sciences, Zoology, Evolutionary biology

## Abstract

Starving animals must balance their resources between immediate survival and future reproduction. False widow spiders, *Steatoda grossa,* inhabit indoor settings with scarce prey. Here, we investigated the effects of lengthy starvation on the physiology, web architecture, sexual signaling, and reproductive success of *S. grossa* females. Compared to well-fed females, starving females (1) lost body mass faster, (2) had lower survival, (3) produced more silk for prey capture than for safety, and (4) deposited less contact pheromone components on their webs but accelerated their hydrolysis to mate-attractant components. As starving females became infecund – but still attracted and copulated with males – they misguided males that would gain reproductive fitness by selecting fecund females. Whether starving females store sperm and potentially regain fecundity upon feeding is still unknown. Our study shows how prey shortage shapes sexual signaling, predation, and reproductive behavior of *S. grossa* females that seem to engage in deceptive signaling.

## Highlights

• Starving unlike fed spiders produced more ‘prey-capture silk’ than ‘safety silk’
• Starving spiders accelerated transitions of contact to mate-attractant pheromones
• Starving females attracted and copulated with males but failed to produce offspring
• Starving females produced dishonest sexual signals not reflecting their infecundity

## Graphical abstract

**Figure:**
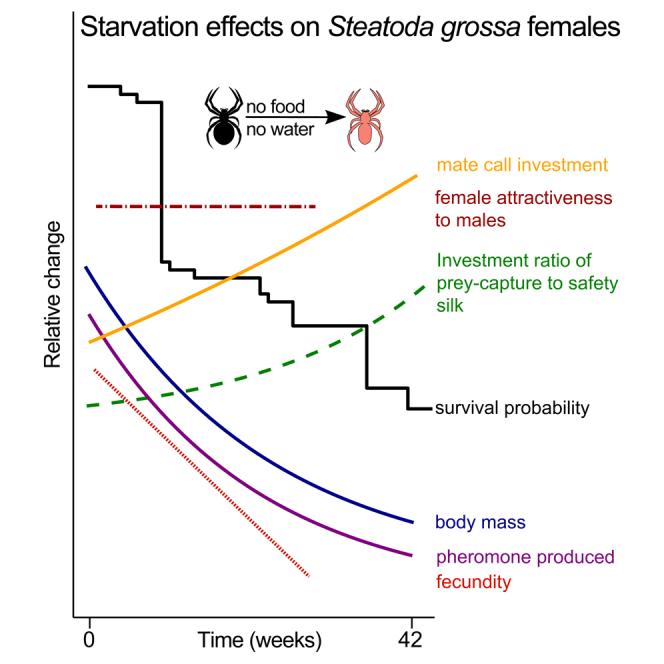

## Introduction

Starving animals must balance their resources (e.g., physical energy and time) between immediate survival and future prospects for reproduction.^1^^,^^2^ For instance, starvation can lead to delayed sexual maturation, decreased mating frequency, and offspring production, while concurrently requiring lower metabolism to conserve resources (reviewed in ^3^). When dire circumstances persist, energy-saving tactics may become detrimental to fitness, setting the stage for the evolution of plastic reproductive strategies to secure mates.^4^

Sexual communication signals between co-evolved senders and receivers help secure mates.^5^ On average, signals accurately represent the ‘state’ of signalers typically aligning with their ‘needs’, whereas rare cheaters communicate based on their ‘needs’ without honestly displaying their poor ‘state’.^6^ Honest signals may be conserved through their high production costs that inferior-quality individuals cannot afford.^7^ This concept is exemplified in the vibrant plumage of male house finches, *Carpodacus mexicanus*, that fades with poor nutrition.^8^ Similarly, the integrity of acoustic, vibratory, and semiochemical signals may diminish under nutritional stress.^9^^,^^10^^,^^11^ It follows that starving signalers might accrue reproductive fitness benefits by producing sexual signals that hide their poor physiological state. Lengthy starvation is particularly costly to unmated females, because their lineage would end without offspring. This risk may prompt terminal investment in reproduction,^12^ even at the cost of accelerating mortality.^13^^,^^14^ Starvation is likely costlier to females than to males, because females bear the costs for producing both large gametes and sexual signals.^15^

The energy costs associated with the production of female sexual signals have hardly been studied despite their relevance in sexual signnaling.^3^^,^^16^ Chemical communication signals are particularly important, as they are used across the animal kingdom,^17^ and are deemed the most ancestral mode of communication.^18^ Contrary to the claim that pheromone production is not expensive,^17^^,^^19^^,^^20^^,^^21^ empirical evidence supporting this claim has been presented in only a single study,^22^ whereas fitness costs – such as reduced lifespan – incurred from pheromone production have been demonstrated in four empirical studies.^10^^,^^23^^,^^24^^,^^25^ High nutritional costs for pheromone production would result in sexual signals affordable only by signalers in prime condition, whereas starved signalers would display their poor condition to prospective mates.

Assessing a prospective mate is adaptive in that the number of offspring produced hinges on the quality of both mates.^26^ Mating with a low-quality partner may reduce the lifetime reproductive output of the high-quality partner.^3^^,^^27^^,^^28^ Additional time and energy costs incur during mate-calling and mate-search^29^^,^^30^ – which can be dangerous^21^^,^^31^ – and during courtship upon mate encounter.^32^^,^^33^ All these costs can be substantial even in promiscuous species,^27^ because resources spent on low-quality mates cannot be recovered. Missed-opportunity costs arise when high-quality mates require costly mate-search.^31^^,^^34^ Lastly, monopolizing reproductive tactics such as mating plugs,^35^ genital mutilation,^36^ or even sexual cannibalism^37^ constrain mate-choice, resulting in fitness costs when the potential partner is a low-quality mate.^38^ In cannibalistic species, the risk of being eaten by a mating partner may be amplified when this partner has been starving.^39^^,^^40^ To attain quality mates, starving signalers that can still afford the signal costs would benefit by hiding their poor physiological state.^6^ Signal recipients, in turn, would benefit from being able to assess the condition of a signaler, such as its hunger state, ideally prior to investing time and energy for locating the signaler. Locating a signaling mate can be particularly challenging for synanthropic species.^41^

Urban or indoor habitats are often characterized by food scarceness, low relative humidity, and fragmented resources, presenting challenges to animals.^42^ Theridiid cobweb spiders commonly occur indoors.^43^ These sit-and-wait predators build their web over long periods of time,^44^^,^^45^ repeatedly adding nutritiously costly proteinaceous silk to their web.^46^ They almost exclusively capture and consume arthropods,^47^^,^^48^ which are relatively scarce indoors,^49^^,^^50^^,^^51^ and are declining in the Anthropocene.^52^^,^^53^ This prey shortage also affects the predators of cobweb spiders, such as other spiders, rodents and lizards.^47^ To survive, cobweb spiders likely trade off safety (the risk of becoming prey) for prey capture (the opportunity of catching prey).^54^

**Figure 1:**
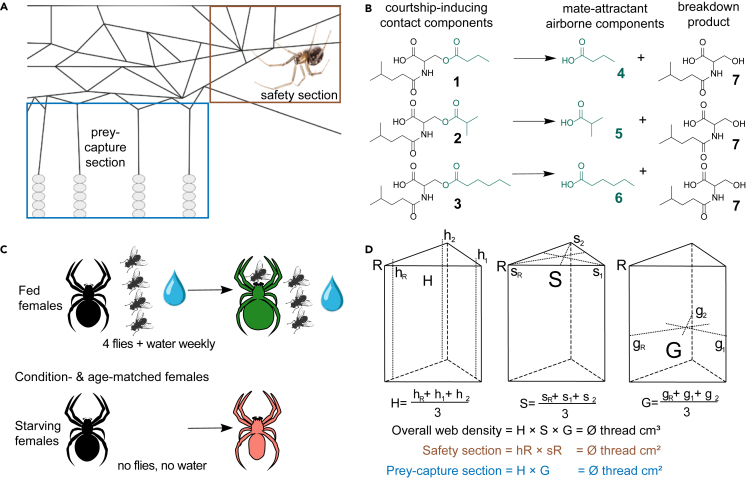
Web architecture and sex pheromone of *Steatoda grossa,* and experimental procedures (A) Cobweb of *S. grossa* female, with distinct safety section (brown) and prey-capture section (blue). (B) Sex pheromone components of female *S. grossa*: three courtship-inducing contact pheromone components [*N*-4-methylvaleroyl-*O*-butyroyl-L-serine (1), *N*-4-methylvaleroyl-*O*-isobutyroyl-L-serine (2), and *N*-4-methylvaleroyl-*O*-hexoyl-L-serine (3)], hydrolyze at the ester bond, and release three corresponding mate-attractant pheromone components [butyric acid (4), isobutyric acid (5), hexanoic acid (6)], whereas the hydrolysis product *N*-4-methylvaleroyl-L-serine (7) accumulates on the web. (C) Experimental spiders: unmated females – matched by age and body condition – were either fed or starved. (D) Web density was measured with a metal rod marked in 1-cm intervals by counting the number of silken strands in each interval. The rod was inserted either vertically 1 cm away from the vertex of the triangular prism in the retreat corner (hR) and the non-retreat corners (h1, h2) of the web, or horizontally at the top of the retreat corner (SR) and the non-retreat corners (s1 and s2) of the triangular prism, pointing to the center of the corresponding hypothenuses. Respective horizontal measurements were taken at the mid-height point of the lateral edges.

Starving predators with an ever increasing need for food trade off safety for prey capture, whereas well-fed animals tend to be risk-averse.^54^ Cobweb-building spiders are fascinating model organisms for studying this trade-off. The webs of widow spiders have distinct regions for safety and prey-capture (Figure 1A).^55^^,^^56^ Glue-impregnated silk strands attached to the ground serve as physical means to entangle pedestrian prey, whereas the retreat corner – characterized by high silk-strand density – provides refuge and protection. Sated females of western black widow spiders, *Latrodectus hesperus,* invested more energy in the safety section of their web, whereas five-day-starved females produced proportionally more prey-capturing silk, revealing need-dependent plasticity in web-architecture.^57^ However, the effect of lengthy starvation on web-architecture and on web-derived signals has yet to be investigated.

Working with unmated females of the false widow spider, *Steatoda grossa* – a close relative of *L. hesperus* – we investigated the effects of lengthy starvation on the females’ physiology, web-architecture, sexual signals, and reproductive success. Mature females are sedentary on their cobweb,^44^ whereas males search for, and are attracted by, the females’ sex pheromone.^58^ Females deposit courtship-inducing contact pheromone components on their web, which then hydrolyze to mate-attractant pheromone components.^59^ Specifically, the contact pheromone components *N-*4-methylvaleroyl*-O-*isobutyrol-L-serine (1), *N-*4-methylvaleroyl*-O-*butyroyl-L-serine (2), and *N-*4-methylvaleroyl*-O-*hexanoyl-L-serine (3) induce male courtship, and enzymatically hydrolyze to the corresponding mate-attractant pheromone components butyric acid (4), isobutyric acid (5), and hexanoic acid (6), all of which readily disseminating from the web, with *N-*4-methylvaleroyl*-O-*serine (7) remaining on the web (Figure 1B). Both the hydrolysis ratio of contact pheromone components and the web-architecture are plastic, being modulated by females in response to perceived same-sex competition.^56^ During courtship, a male bundles up the female’s web, thereby increasing his chances to copulate.^60^ Sexual cannibalism may occur for 5% of males.^60^ Globally distributed, *S. grossa* is commonly found indoors,^61^ but ancestrally may have inhabited caves.^62^ That *S. grossa* apparently withstands several weeks of starvation without noticeable physiological changes^63^ implies evolutionary adaptations to habitats with periodic and sustained food shortages. Females may live for several years, whereas males die within a few months of maturation.^64^^,^^65^

Considering the extended lifespan of *S. grossa* and its apparent adaptation to lengthy starvation,^63^ and building on the need-perceived plasticity of sexual signaling and web-architecture in *S. grossa,*^56^ we tested four hypotheses: starving females (1) lose weight faster and die sooner than fed females, (2) produce lower-density webs with more prey-catching gum-footed lines, (3) adjust the deposition of courtship-inducing contact pheromone components and their hydrolysis to mate-attractant pheromone components, and (4) produce dishonest sexual signals not reflecting their poor fecundity.

## Results

### Hypothesis 1: Starving females lose weight faster and die sooner than fed females

**Figure 2:**
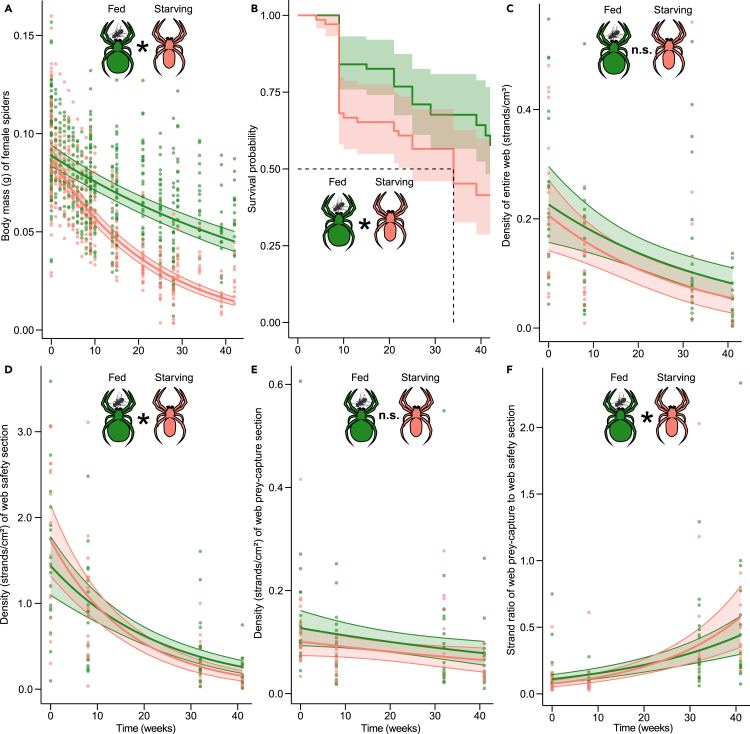
Effects of starvation on the physiology and web architecture of unmated female *Steatoda grossa* (A) Changes in body mass over time of 69 fed and 69 starving females. (B) Survival probability (Kaplan-Meier plot) of 69 fed and 69 starving females. (C) Changes in silk strand density of 38 entire webs. (D) Changes in numbers of silk strands/cm^2^ in the web safety section of 38 webs. (E) Changes in numbers of silk strands/cm^2^ in the web prey-capture section of 38 webs. (F) Silk strand ratio of the web prey-capture section to the web safety section of 38 webs. Green- and red-colored symbols represent data of individual fed and starving females, respectively. Solid lines represent curves fitted from generalized linear mixed models, with shaded areas indicating 95% confidence intervals for panels A, and C–F. An asterisk (∗) denotes a statistically significant difference between data of starving and fed females; ‘n. s.’ = not significant.

Starvation significantly reduced the body mass of *S. grossa* females. Both starved females (*N* = 69) and fed females (*N* = 69) lost body mass steadily over 42 weeks (GLMM, *N* = 138, χ^2^ = 128.00, df = 1, *p* < 0.001, Exp. 1, Figure 2A) but food- and water-deprived females lost body mass significantly faster (GLMM, *N* = 138, χ^2^ = 138.49, df = 1, *p* < 0.001, Exp. 1, Figure 2A). This differential loss of body mass between starved and fed females took effect only after 12 weeks (Tukey post hoc test, z = 7.96, *p* < 0.001).

Starving females had a 71% greater mortality risk than fed females (COX-PH, *N* = 138, χ^2^ = 4.09, df = 1, *p* = 0.04, Exp. 2, Figure 2B). At week 34, >50% of starving females were dead, whereas 58% of fed females were still alive at week 42 when the experiment was terminated (Figure 2B).

### Hypothesis 2: Starving females produce lower-density webs with more prey-catching gum-footed lines

The density of webs (silk strands per cm^3^ of web) declined over time (GLMM, *N* = 38, χ^2^ = 15.33, df = 1, *p* < 0.001, Exp. 3, Figure 2C), with similar declines across the webs of 19 starving and 19 fed spiders (GLMM, χ^2^ = 0.58, df = 1, *p* = 0.446, Exp. 3, Figure 2C). Likewise, over time there were fewer silk strands in the safety section of webs (GLMM, *N* = 38, χ^2^ = 8.94, df = 1, *p* < 0.001, Exp. 3, Figure 2D). Reduced investment in ‘safety silk’ was more evident in webs of starving females than fed females (GLMM, χ^2^ = 4.57, df = 1, *p* = 0.032, Exp. 3, Figure 2D). All females produced fewer prey-catching gum-footed lines over time (GLMM, *N* = 38, χ^2^ = 5.72, df = 1, *p* = 0.016, Exp. 3, Figure 2E), with no differential effect between starving and fed females (GLMM, χ^2^ = 0.02, df = 1, *p* = 0.894, Exp. 3, Figure 2E). For all spiders, however, there was a trade-off between predation and protection (the opportunity of catching prey versus not becoming prey) (GLMM, *N* = 38, χ^2^ = 48.99, df = 1, *p* < 0.001, Exp. 3, Figure 2F). Compared to fed spiders, starving spiders produced relatively more silk strands for prey capture than for protection (GLMM, χ^2^ = 4.43, df = 1, *p* = 0.035, Exp. 3, Figure 2F).

### Hypothesis 3: Starving females adjust the deposition of courtship-inducing contact pheromone components and their hydrolysis to mate-attractant pheromone components

**Figure 3:**
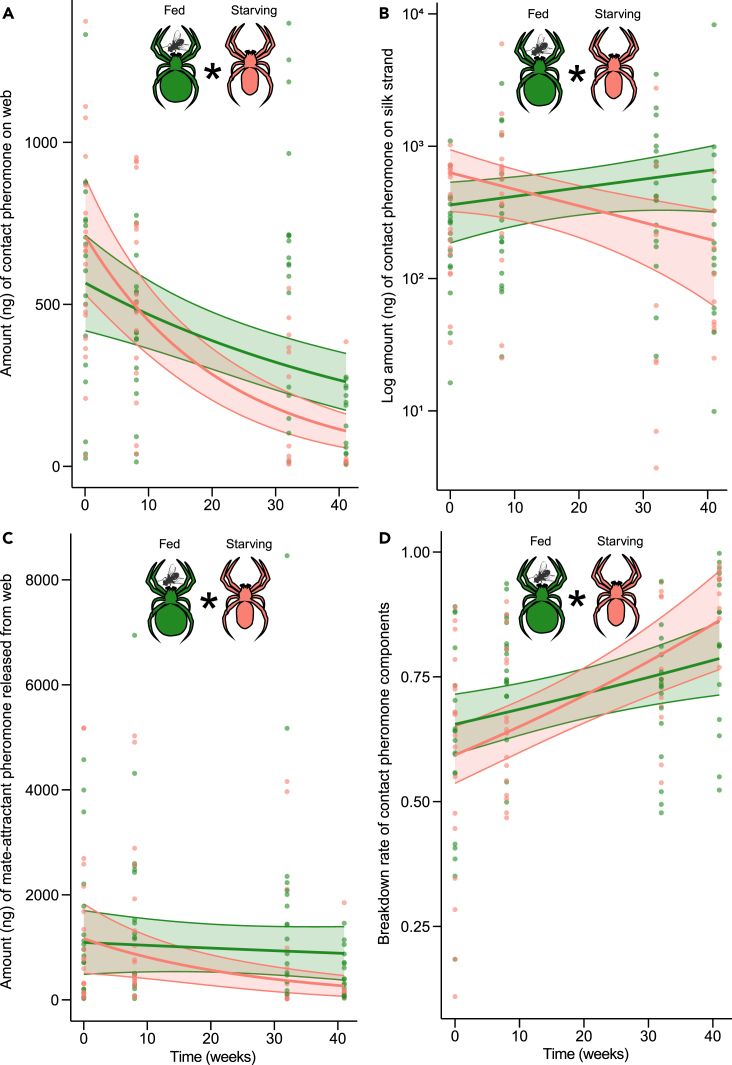
Effects of starvation on sex pheromone production of unmated female *Steatoda grossa* (A) Amount of contact pheromone components deposited by 38 females on their webs. (B) Amount of contact pheromone components per silk strands of 38 webs. (C) Amount of mate-attractant pheromone components released from a web. (D) Breakdown ratio of contact to mate-attractant pheromone components. The green- and red-colored symbols represent data of individual fed and starving females, respectively. Solid lines represent curves fitted from generalized linear mixed models, with shaded areas indicating 95% confidence intervals. An asterisk (∗) denotes a statistically significant difference between data of starving and fed females.

Starvation had a strong effect on the amount of contact pheromone components that females deposited on their webs (GLMM, *N* = 38, χ^2^ = 14.98, df = 1, *p* < 0.001, Exp. 4, Figure 3A). Over time, starving females deposited less pheromone than fed females (GLMM, χ^2^ = 11.41, df = 1, *p* < 0.001, Exp. 4, Figure 3A). When the amount of pheromone was normalized on the number of silk strands per web, fed females deposited more pheromone over time, whereas starving females deposited less (GLMM, χ^2^ = 11.18, df = 1, *p* < 0.001, Exp. 3, Figure 3B). Similarly, webs of starving females, but not of fed females, released lower amounts of mate-attractant pheromone components over time (GLMM, χ^2^ = 5.58, df = 1, *p* = 0.018, Exp. 4, Figure 3C).

As starved and fed females aged, they increased hydrolysis of contact pheromone components on their webs to mate-attractant pheromone components (GLMM, *N* = 38, χ^2^ = 8.803, df = 1, *p* < 0.001, Exp. 4, Figure 3D). This increase in contact pheromone hydrolysis was greater in starving females (*N* = 19) than in fed females (*N* = 19) (GLMM, χ^2^ = 4.03, df = 1, *p* = 0.045, Exp. 4, Figure 3D).

### Hypothesis 4: Starving females produce dishonest sexual signals not reflecting their poor fecundity

**Figure 4:**
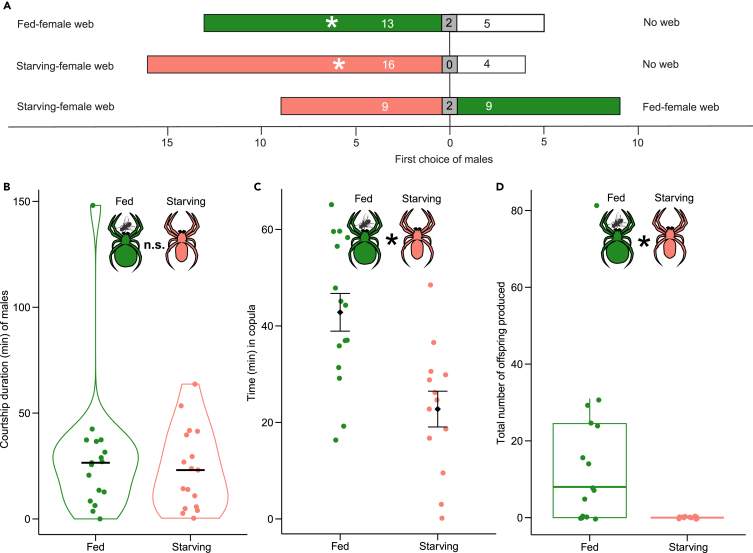
Effects of starvation on unmated female *Steatoda grossa* on their ability to secure a mate and on their lifetime reproductive success (A) Attraction of males to webs of starving and fed unmated females; central gray boxes represent non-responders. (B) Violin plots indicating the duration males courted on webs of fed and starving females, with the horizontal bar representing the median. (C) Time that fed and starving females stayed *in copula*; black squares and whiskers represent the mean and standard error. (D) Boxplots showing the number of viable offspring produced by fed and starving females during their life. In B, C, and D, colored symbols represent data of individual bioassay replicates. An asterisk (∗) denotes a statistically significant difference between data of starved and fed females; n. s. = not significant.

Starvation (28 weeks) of female spiders did not diminish their webs’ attractiveness to males. Frames bearing the webs of fed females (*N* = 19; Exp. 5) and starved females (*N* = 19; Exp. 6) were more attractive to males than empty frames (Exp. 5: 13 vs*.* 5; binomial test, *p* = 0.048; Exp. 6: 16 vs. 4; binomial test, *p* = 0.006, Figure 4A). When males were given a choice between frames bearing the web of a starving female or a fed female, they exhibited no preference (Exp. 7: 9 vs. 9; binomial test, *p* = 1.00, Figure 4A), even though webs of females starving for 28 weeks released 20% less pheromone than webs of fed females (Tukey post hoc test, z = 2.10, *p* = 0.035).

Starvation did not affect the females’ ability to secure a mate, but it did affect their lifetime reproductive success. The extent of time males courted on webs of fed and starving females did not differ (U-Test, N_fed_ = 17, N_starved_ = 17, W = 154, *p* = 0.757, Exp. 8, Figure 4B), even though at week 28 webs of starving females bore 53% less contact pheromone components than webs of fed females (Tukey post hoc test, for entire webs: z = 2.61, *p* = 0.009; for silk strands: z = 2.05, *p* = 0.040). Fifteen fed females and 13 starving females copulated (Fisher test, *N* = 34, *p* = 0.656, Exp. 8, Figure 4C). Fed females stayed *in copula* twice as long as starving females (t-test, t = 3.72, df = 25.99, *p* < 0.001, Exp. 8, Figure 4C). Only a single male was cannibalized by a starving female. Unlike fed females, starving females – despite copulation – failed to produce offspring (U-Test, N_fed_ = 17, N_starved_ = 17, W = 162.5, *p* < 0.001, Exp. 8, Figure 4D).

## Discussion

Having starved for a long time, unmated *S. grossa* females still engaged in sexual signaling and attracted males despite their complete loss of fecundity. Starving females produced as much silk as fed females but changed their web’s architecture, in that they produced more ‘prey-capture silk’ than ‘safety silk’. Males were equally attracted to webs of starving and fed females and courted equally long on either type of web. However, males stayed *in copula* with fed females twice as long as with starving females. The incidence of sexual cannibalism did not increase with starvation. Our study demonstrates the ability of *S. grossa* to withstand extreme starvation stress and it suggests deceptive signaling by females.

### Hypothesis 1: Starving females lose weight faster and die sooner than fed females

We had predicted that starving females suffer significant losses in body mass and experience a shorter life than fed females, but we were surprised to find that effects of starvation became apparent not before 12 weeks after experiment initiation, with many spiders still being alive after 42 weeks of starvation. Obviously, false widow spiders are remarkably well adapted to periodic or sustained food shortages in the urban environment they inhabit. That the growth trajectory of developing *S. grossa* slows during prey scarcity^66^ is yet another adaptation to cope with prey shortages. Similarly, synanthropic redback spiders, *Latrodectus hasselti*, survive nearly six months without food,^67^ but western black widows, in contrast, exhibit physiological distress just one week after food deprivation.^57^ Resilience to starvation is common among urban arthropods, such as bed bugs, *Cimex lectularius*, or cockroaches, that can withstand lengthy starvation.^68^^,^^69^ The mechanisms enabling lower metabolic rates and energy conservation during starvation are well documented,^2^^,^^3^^,^^70^ particularly for ectoparasites such as bed bugs and ticks.^71^^,^^72^ Predators, including web-building spiders, depend on the presence of arthropod prey, which tend to be scarcer in human dwellings than in natural landscapes.^49^^,^^50^^,^^51^^,^^52^^,^^53^ Limited prey availability in urban habitats affects not only spiders but also vertebrate predators, such as great tits, *Parus major*, that in response to prey shortage produce smaller broods with lower nestling survival.^73^ Lack of food forces animals to abandon risk-averse behavior in favor of risk-taking behavior with better chances of finding food.^54^

### Hypothesis 2: Starving females produce lower-density webs with more prey-catching gum-footed lines

Foraging behavior of animals without becoming prey themselves is based on a state-dependent cost-benefit trade-off.^74^ Natural selection favors behavior that helps secure food in the safest manner possible. Foraging squirrels, e.g., favor lower-value safe food to higher-value risky food.^75^^,^^76^ The starvation-predation hypothesis states that risk-prone behavior is favored when the threat of starvation supersedes the danger of becoming prey.^77^^,^^78^ This hypothesis is supported by the plastic behavior of sessile cobweb spiders. When hungry, females of *L. hesperus* and the bell-shaped cobweb spider, *Campanicola campanulata,* produce more prey-capture silk than ‘safety’ silk for their webs’ retreat.^57^^,^^79^ Conversely, well-fed female *C. campanulate* triple the silk volume in their retreat.^79^

Our findings support the starvation-predation hypothesis in that starving *S. grossa* females increased the ratio of prey-capture silk to safety silk more quickly than fed females (Figure 2F). However, starving females did not produce more prey-capture silk (Figure 2E) but – instead – produced less safety silk for their webs’ retreat (Figure 2D), maintaining a similar overall web density as fed females (Figure 2C). As prey-capture silk contains nutrient-rich glue^80^ and unfed females are nutrient-starved, this conservative investment in prey-capture silk seems prudent. It is somewhat perplexing, though, that starved females kept an overall high web density (Figure 2C) because silk production incurs high nutrient costs,^46^ and web production costs are particularly high for cobweb spiders which do not consume silk, and continually extend their web throughout their life.^45^ Females of *S. grossa* even appropriate pre-existing web-like structures to save costs associated with building a web *de novo*.^44^^,^^81^ The silk strand density of webs might also serve as an honest signal to males that make contact with the entire web during their web-reducing courtship.^60^

### Hypothesis 3: Starving females adjust the deposition of courtship-inducing contact pheromone components and their hydrolysis to mate-attractant pheromone components

Sex pheromone production by starving female *S. grossa* was significantly compromised. Compared to fed females, starving females lowered production and deposition of contact pheromone components on their web (Figure 3A) that prompt courtship by males and that hydrolyze to mate-attractant pheromone components (Figure 3C). However, starving females compensated for diminished amounts of contact pheromone components by increasing their hydrolysis ratio to mate-attractant pheromone components (Figure 3D), essentially maintaining their web’s attractiveness to mate-seeking males (Figure 4A). These data imply substantial energetic costs associated with pheromone production, and they unravel mechanisms that help starving females stay attractive to males and hide their poor physiological state.

Production costs of the contact pheromone components 1, 2, and 3 are deemed high because they contain nitrogen and are biosynthesized through amino acid metabolism.^82^^,^^83^ This concept of costly pheromone biosynthesis by widow spiders was first invoked for the sex pheromone of female *L. hesperus*^40^ that produce sex pheromone components structurally similar to those of *S. grossa*.^reviewed in 83^ Silk extract of starving female *L. hesperus* prompted less ‘activity’ by conspecific males than silk extract of fed females, indicative of quantitative differences in deposited and extracted pheromone.^40^ In our study, female spiders curtailed contact pheromone deposition in response to starvation – implying significant pheromone production costs – but the success of this cost-saving measure hinges on the response of conspecific males.

### Hypothesis 4: Starving females produce dishonest sexual signals not reflecting their poor fecundity

Webs of starving female *S. grossa* remained as attractive to mate-seeking males as webs of fed females (Figure 4A). Even though starving females were in poor condition (Figure 1A), and not fecund (Figure 4D), their web-derived pheromone signals misinformed mate-selection by males that would gain reproductive fitness by selecting – instead – fecund females in prime physiological condition. Starving females achieved this deception by accelerating the hydrolysis of contact pheromone components to mate-attractant pheromone components, thereby compensating for the diminished amount of contact pheromone components they were able to deposit on their web (Figures 3A and 3C). Upon arrival on webs, males still courted starving females as much as fed females (Figure 4B), even though webs of starving females bore 53% less contact pheromone components. The males’ indiscriminate responses suggest that deposits of contact pheromone components on individual silk strands (Figure 3B) were still sufficient to not lessen courtship. This interpretation is in line with previous findings that male *S. grossa* reduced courtship efforts only when the concentration of contact pheromone decreased by more than 10-fold.^59^

Males copulated with starving unmated females as readily as with fed unmated females (Figure 4C), suggesting that the poor physiological condition of starving females was not apparent to males. In some arthropods, however, starving females are not as readily mated, or – alternatively – they accept mates less often.^2^^,^^3^ For example, hungry females of *Gerris buenoi* water striders are less ready to mate than satiated females.^84^ That starving female *S. grossa* stayed *in copula* much shorter than fed females (Figure 4C) parallels finding in others insects.^2^^,^^3^ For example, female small water striders, *Microvelia austrina,* copulate for shorter periods in response to starvation.^85^ In *S. grossa,* shorter copulation times do not necessarily reflect diminished sperm transfer. In many theridiid spiders, sperm is transferred mostly at the beginning of copulations,^86^ inspiring studies to investigate the biological significance of lengthy or repeated copulation in spiders.^87^ In our study, we did not test whether males transferred sperm to starving females, and we did not investigate whether short copulations were the female’s or the male’s decision.

Failure to produce any offspring was the detrimental impact that lengthy starvation exerted on the reproductive fitness of *S. grossa* females. Their sexual communication signals misguided conspecific males and lowered their potential reproductive fitness. Males that cannot assess the quality of a signaling female may incur significant costs. Orienting toward webs of infecund females, males are at risk of falling prey in the process, and they forgo opportunities for prey-capture.^65^ Moreover, when males engage in elaborate courtship with infecund females, bundling up their web whilst adding their own silk,^60^ they invest significant time and energy, and they expend nutrients with their proteinaceous silk that they add to the females’ web.^33^^,^^46^ Lastly, when the short-lived males court infecund females, they miss opportunities to secure high-quality fecund females that would ensure the males’ reproductive success. That 67% of fed females produced offspring indicates the need for prey nutrients in order to reproduce, as also shown in the wolf spider *Pardosa pseudoannulata*.^88^

The benefits that starving *S. grossa* females accrue by deceptively attracting conspecific males, and by copulating with them, remain unknown. Starving females do not seem to lure males to consume them, because only one male in 34 pairs with a starving or a well-fed female was cannibalized. Generally, though, starving female spiders and mantids consume their mates more often than fed females.^3^ Similarly, males of *L. hasselti* were more likely to be eaten by starving than by fed females,^89^ and males of *L. hesperus* avoided hungry females.^40^ Female *S. grossa* also incur nutritional and opportunity costs when they are courted by a male who cuts and bundles up sections of the female’s web. Following courtship, the female then needs to rebuild her web,^33^ which takes time and energy, and results in delayed or even missed prey-capture opportunities. In insects, nutrient transfer can accompany sperm transfer^90^^,^^91^ but this has not yet been demonstrated in spiders. Alternatively, mated spider females may store sperm for use as soon as they have captured and consumed enough prey and have become fecund again. Even if this were shown, these females would be inferior mates to males because their reproductive output is delayed and uncertain. Adverse effects of delayed reproductive output are likely amplified by reproductive senescence.^92^

Aging during the 42-week experiment affected all spiders, irrespective of whether they were fed or starving. Even food-provisioned spiders lost significant weight, and 42% died. Over time, both fed and starving spiders produced and deposited less silk and less pheromone. Conceivably, aging itself may constrain the spiders’ ability to build new webs, or repeatedly building new webs may incur significant costs. How aging affects the signaling and reproductive strategy of unmated female *S. grossa* is currently being investigated.

### Limitations of the study

We studied the combined effects of food- and water-deprivation on sexual signaling and the reproductive ability of *S. grossa* females but did not isolate the effect of either food or water on its own. Furthermore, whereas mated females were completely infecund after 28 weeks of starvation, it is not clear when females became infecund, and whether they gradually or abruptly lost fecundity. Lastly, it remains to be investigated whether starving females regain fecundity following prey captures and replenishment of their nutritional resources.

### Conclusion

In conclusion, our study unravels a complex interplay between starvation, reproductive fitness (or lack thereof), sexual signaling, and the trade-off between predation and safety (the opportunity of catching prey versus not becoming prey) in the false widow spider *Steatoda grossa*. Widow females were remarkably resilient to starvation reflecting their adaptation to enclosed environments with periodic and lengthy prey shortages. Starving females engaged in strategic signaling. They lowered production and deposition of contact pheromone components (suggesting costly biosyntheses) but accelerated their transition to mate-attractant pheromone components. Starving females altered their web-architecture, allocating more silk for prey-capture without affecting the overall web-density. Surprisingly, starving females remained as attractive as well-fed females and prompted courtship and copulation by males. As starving females became infecund, they deceived mate-selection by males that would gain reproductive fitness by selecting – instead – fecund females as mates. By deceptively attracting and mating conspecific males, starving females may receive sperm that they could use as soon as they have captured enough prey and have become fecund again. Nonetheless, these females would still be inferior mates because their reproductive output is delayed and uncertain. Our study shows how prey shortage – as an environmental stressor – shapes sexual signaling as well as reproductive and predation behavior, and it suggests deceptive signaling in a synanthropic spider.

## Resource availability

### Lead contact

Further information and requests should be addressed to the corresponding author, Andreas Fischer (andreas.fischer@uni-greifswald.de).

### Materials availability

This study did not generate novel reagents.

### Data and code availability

• All data [S1] are available in Supplementary Information.
• The complete R code [S2] is available in Supplementary Information.
• All other data supporting the findings of this study are available within the paper and its Supplementary Information.
• Any additional information required to reanalyze the data reported in this paper is available from the lead contact upon request.

## Acknowledgments

We thank Gabriele Uhl for valuable discussions on the manuscript, and four anonymous Reviewers for constructive comments. A.F. was supported by an Alexander Graham Bell Scholarship from the 10.13039/501100000038Natural Sciences and Engineering Research Council of Canada (NSERC), Graduate Fellowships from SFU, the H.R. McCarthy Bursary, and by an Oscar and Jan Francke Student Research Fund of the International Society of Arachnology. Funding for this study was provided by an NSERC - Industrial Research Chair to G.G., with BASF Canada Inc. and Scotts Canada Ltd. as the industrial sponsors.

## Author contributions

Conceptualization, A.F.; Methodology, A.F.; Investigation, A.F., N.D.V., and S.P.S.; Data Curation, A.F.; Writing – Original Draft, A.F; Writing – Review and Editing, G.G. and A.F.; Funding Acquisition, G.G.; Resources, G.G. and A.F.; Supervision, A.F.

## Declaration of interests

The funders (NSERC, BASF Canada Inc., Scotts Canada Ltd.) had no role in the study design, data collection and analysis, decision to publish, or preparation of the manuscript.

## STAR★Methods

### Key resources table

**Table:** 

REAGENT or RESOURCE	SOURCE	IDENTIFIER
**Chemicals, Peptides, and Recombinant Proteins**		

*N-*4-methylvaleroyl*-O-*isobutyrol-L-serine	Synthesized in our lab for a previous study	https://doi.org/10.1038/s42003-022-04072-7
*N-*4-methylvaleroyl*-O-*butyroyl-L-serine	Synthesized in our lab for a previous study	https://doi.org/10.1038/s42003-022-04072-7
*N-*4-methylvaleroyl*-O-*hexanoyl-L-serine	Synthesized in our lab for a previous study	https://doi.org/10.1038/s42003-022-04072-7
*N-*4-methylvaleroyl*-O-*serine	Synthesized in our lab for a previous study	https://doi.org/10.1038/s42003-022-04072-7

**Deposited Data**		

All data and code are presented in this manuscript	Supplementary materials	

**Experimental Models: Organisms/Strains**		

*Steatoda grossa*	Collected in building hallways of Simon Fraser University, Burnaby, Canada	

**Software and Algorithms**		

R Studio	https://www.r-studio.com/	R Core Team (2022). R: A language and environment for statistical computing (R Foundation for Statistical Computing).

### Experimental model and study participant detail

Specimens of *S. grossa* used in our study were the F3 and F4 offspring of spiders collected on the Burnaby campus of Simon Fraser University (49°16′44″*N* 122°54′58″W).^93^ The spiders were kept singly in the insectary maintained at 22°C, about 38% relative humidity, and a reverse photocycle (12 h L: 12 h dark), resembling the hallway settings from which spiders were collected. Juvenile spiders were kept in Petri dishes (100 × 20 mm) and fed *Drosophila melanogaster* vinegar flies, whereas mature females were kept in clear 300-mL plastic cups (Western Family, Tigard, Oregon, USA) and fed four *Phormia regina* blow flies per week, with water supplied on cotton. The size of spiders was determined based on (*i*) the average tibia-patella length of the first leg-pair measured using ImageJ (The National Institute of Health, Gaithersburg, USA), and (*ii*) smartphone photographs with a microscope adapter. Spiders were weighed using a TR-204 Denver Instrument Company Scale (Denver Instruments, Arvada, Colorado, USA). The body condition of spiders was determined from the residuals of a linear model based on body mass and size.^94^ The post sexual maturity age of the 138 test spiders ranged between 119 and 720 days with a median of 292 days, whereas the body condition ranged between −0.05 and +0.05, with a median of 0.0002.

### Method details

#### Setting up treatment groups

To examine the effects of food- and water-deprivation (henceforth ‘starvation’) on the females' sexual signals and fecundity, unmated females – matched by age and body condition – were assigned to a ‘starved’ and a ‘fed’ treatment group (Figure 1C). Starved-group spiders were food- and water-deprived throughout the 42-week experiment, whereas fed-group spiders were food- and water-provisioned as described above. The mean age differential (days post sexual maturity) between pairs was 0.48 ± 0.37, and the mean body condition differential was 0.0001 ± 0.00049.

#### Assessment of spider body mass and longevity

To assess the effect of starvation on the body mass of unmated females, fed and starved females were weighed over the course of 42 weeks (Exp. 1). In spring 2021, 38 spiders were weighed singly every week for 10 weeks, followed by six additional weight-measurements extending to 42 weeks of starvation. These 38 females were used for web-measurement (Exp. 3), and for pheromone quantifications (Exp. 4). In fall 2021, 50 females each were added to the groups of starved females and of fed females, and were weighed 6-times over 28 weeks, keeping the experimentalist blind to the treatment (here, and throughout all experiments in the study). The 100 spiders we added during the fall were subsequently used for mate-choice and mating trials (Exps. 5–8). Mortality caused by starvation (Exp. 2) was assessed by inspecting the 69 fed and the 69 starved spiders weekly over 42 weeks.

#### Web measurements

To assess spider starvation effects on web architecture, the web density of 19 fed and 19 starved unmated females was measured on weeks 0, 8, 33, and 42. To enable construction of an entirely new web, each female was placed on a triangular bamboo frame (18 × 18 × 25 cm) and allowed three days to build a web.^93^

Web density was quantified^56^^,^^95^ by counting silk strands at each 1-cm interval on a metal rod inserted along nine specific axes within the web (Exp. 3; Figure 1D). Measurements were taken on (*i*) vertical axes (H) near both retreat (hR) and non-retreat corners (h1, h2), (*ii*) top horizontal axes (S) from both retreat (sR) and non-retreat corners (s1, s2) across the web, and (*iii*) mid-horizontal axes (G) from corners (gR, g1, g2) across the web. Vertical (H) and horizontal measurements (S, G) consisted of 22 and 15 1-cm intervals, respectively. To avoid multiplication with zero, averages were calculated after a value of one was added to a single increment of each of the nine measurements.

The overall web density is the product of the averages from vertical (H), top horizontal (S), and mid-horizontal (G) axes, revealing silk strands per cubic centimeter. Silk strand density in the retreat section of the web was estimated as the product of the retreat vertical (hR) and retreat top horizontal (sR) measurements, whereas the prey-capture section of the web was estimated as the product of the vertical (H) and mid-horizontal (G) measurements. The investment ratio between ‘prey-capture silk’ and ‘safety-silk’ was calculated to assess the proportional impact of starvation on web architecture.

#### Quantification of the females’ sex pheromone

Starvation-caused quantitative changes in contact pheromone components deposited by females on their web (Exp. 4) were assessed for the four webs (produced in weeks 0, 8, 33, and 42) of each of the 38 females used in experiment 3. Each density-measured web was extracted 24 h in 50-μL methanol (99.9% HPLC grade, Fisher Chemical, Ottawa, Canada), and aliquots (2 μL) of extracts were injected into a Bruker maXis Impact Quadrupole Time-of-Flight LC/MS (liquid chromatography–mass spectrometry) system. The Spursil C18 column (30 mm × 3.0 mm, 3 microns; Dikma Technologies, Foothill Ranch, CA, USA) was heated to 30°C and eluted with a solvent gradient (0.4 mL/min), starting with 80% water and 20% acetonitrile, and ending – after 4 min – with 100% acetonitrile. The solvent system contained 0.1% formic acid to enhance the peak shape of compounds. The mass spectrometer was set to positive electrospray ionization (+ESI) with a gas temperature of 200°C and a gas flow of 9 L/min. The nebulizer was set to 4 bar and the capillary voltage to 4200 V.

Pheromone components were quantified using external synthetic standards. The mate-attractant pheromone components were quantified using 7 as a proxy for 4, 5, and 6, because both 7 and the sum of 4, 5, and 6 (Figure 1B) have equal stoichiometric quantities.^56^^,^^59^^,^^83^ The hydrolysis ratio of contact pheromone components and the corresponding release rate of mate-attractant pheromone components were calculated as 7/(1 + 2 + 3 + 7). Using this method, the amount of contact pheromone components was quantified for each of the four webs. However, as a male spider courting on a web makes contact with single silk strands, the amount of contact pheromone components on each web was divided by its silk strand density to estimate the amount of contact pheromone components per silk strand.

#### Attraction of males to webs of fed and starved females in Y-tube olfactometers

Attraction of *S. grossa* males to webs of fed or starved females was tested in Y-tube olfactometers.^60^ To prepare test stimuli, females – matched by age and body condition – were grouped in 40 pairs, with one female in each pair being fed and the other starved. After 28 weeks, the females in each pair were placed on separate bamboo frames (see above) to build a web for three days. Each frame – together with the freshly-spun web and the female spider – was placed in a translucent oven bag (30 cm × 31 cm; Toppits, Mengen, DE), which was secured to the opening of a Y-tube side-arm. To initiate a bioassay, a naive male was placed into a holding tube (26 cm long; 2.5 cm diameter) and allowed 2 min to acclimatize. The holding tube was then attached to the Y-tube olfactometer (main stem: 24 cm long, side arms 21 cm long, all 2.5 cm diam.), and air was drawn at 200 mL/min through the olfactometer. Single males were offered a choice between (*i*) a fed female on her web vs. an empty frame (*N* = 20, Exp. 5), (*ii*) a starved female on her web vs. an empty frame (*N* = 20, Exp. 6), and (*iii*) a fed female on her web vs. a starved female on her web (*N* = 20, Exp. 7). A response was recorded when the male entered one of the two oven bags within 5 min. Thus, choices were based solely on olfaction prior to contact with the female’s web. After bioassay replicates, oven bags and content (excluding spider males) were discarded, and the olfactometer and holding tube were washed with soapy water and dried 3 h in an oven at 100°C.

#### Lifetime reproductive success of fed and starving females

Mating trials further assessed the effect of starvation on the females’ ability to secure mates, and on their lifetime reproductive success. Each of 17 starving females and 17 fed females was paired with the very same male (Exp. 8) who was previously attracted to the female’s web in Y-tube olfactometer bioassays (Exps. 5–7). In each bioassay replicate, the bamboo frame supporting the female’s web – with the female residing on the web – was placed into an acrylic chamber (15 cm × 15 cm × 20 cm), a male was added, and the pair’s behavior was video-recorded for 4 h (Handycam, HDR-XR550; Sony, Tokyo, Japan). Videos were analyzed using VLC player (VideoLAN, Paris, France) to quantify the courtship duration of the male, as well as the incidence and duration of a copulation. Females that copulated were deemed mated and were starved, and their lifetime total number of offspring produced was tracked.

### Quantification and statistical analysis

Data were analyzed and visualized using R.^96^ Results are presented as means ± standard errors of the mean, or 95% confidence interval of models. The number of independently run replicates (N) is stated in figure captions, and at least 17 replicates were run in all experiments. The threshold for significance was *p* < 0.05.

Decline in body mass (Exp. 1) was analyzed with a generalized linear mixed model (glmmTMB, Tweedie distribution), with body mass as the dependent variable, and week (time) and treatment (fed or starved) as fixed effects. A random intercept for each spider accounted for repeated measures, and accommodated mass variability of individual spiders over time. An additional random intercept for each cohort (spring or fall) accounted for potential variability between these cohorts. Differential model slopes were assessed with a type III ANOVA (Anova, car package). The onset of differential body mass due to starvation was identified through post-hoc Tukey-corrected pairwise comparison (emmeans package). Survival rates of spiders (Exp. 2) were compared using the Cox Proportional Hazard model (COX-PH, survival package), with Kaplan-Meier survival plots visualizing survival probabilities over time.

The effect of starvation on web architecture (Exp. 3) was analyzed using generalized mixed effect models, following the model structure described above (Exp. 1).

The effect of starvation on pheromone signals (Exp. 4) was analyzed by generalized mixed effect models (as outlined above), except that a negative binomial (nbinom2) distribution was used for pheromone quantities.

Choice data in experiments 5 and 6 were analyzed using a one-way binomial test, whereas choice data in experiment 7 were analyzed using a two-way binomial test.^59^

Mann-Whitney-U-Tests were used to compare male courtship durations between treatments, as well as the number of viable offspring produced throughout the life of the mothers. The incidence of copulations and sexual cannibalism across treatments were each compared using Fisher exact tests. Moreover, the duration of copulation was compared between fed and starving females using a Welch two sample T-test.

## Supplemental information

Table S1. Raw data from all experiments of the study and basis to generate figures 2–4, too large to fit in a PDF, related to STAR Methods

Document S1. Code in R used to analyze raw data S1, related to STAR Methods
