# Supplementary material for: Starving infecund widow spiders maintain sexual attractiveness and trade off safety for enhanced prey capture
Source: iScience. 2024 Aug 13;27(9):110722. doi: 10.1016/j.isci.2024.110722 (PMC11396026; doi:10.1016/j.isci.2024.110722)
Supplement: Document S1. Code in R used to analyze raw data S1, related to STAR Methods [file mmc2.pdf]

```
rm(list=ls())
#load libraries
library(ggplot2)
library(readxl)
library(emmeans)
library(glmmTMB)
library(car)
library(DHARMa)
library(dplyr)
library(survival)
library(survminer)
#Include all females (web measurement and y tube)
starv_mass <- read_excel("starvationdata.xlsx",
                    sheet = "mass")
starv_mass$week<-as.numeric(starv_mass$week)
starv_mass$mass<-as.numeric(starv_mass$mass)
starv_mass$treatment<-as.factor(starv_mass$treatment)
starv_mass$spider<-as.character(starv_mass$spider)
View(starv_mass)

# sample size and duration of treatments
starv_mass %>%
  group_by(spider) %>%
  summarise(count = n(), .groups = 'drop') %>%
  count(count) #counts = number of weighing, n = sample size for each frequency
#glmmTMB starvation model
weektreatment <- glmmTMB(mass ~ week * treatment + (1|cohort/spider) + (week|spider),
                    data = starv_mass,
                    family = tweedie(),
```

```
                    na.action = na.exclude)

weektreatment0 <- glmmTMB(mass ~ week*treatment + (week|spider), data = starv_mass, family =
tweedie(), na.action = na.exclude)

anova(weektreatment0, weektreatment) #test whether the random factor cohort has an effect on
the model

summary(weektreatment)

Anova(weektreatment,type="III")


#test residuals

residuals_df <- data.frame(residuals = resid(weektreatment))

ggplot(residuals_df, aes(x = residuals)) +

  geom_histogram(bins = 30, fill = "blue", color = "black") +

  labs(title = "Model Residuals", x = "Residuals", y = "Frequency")

plot(residuals(weektreatment) ~ fitted(weektreatment), main="Residuals vs Fitted")


#posthoc test to identify latency till mass difference between the treatments:

emm_interaction <- emmeans(weektreatment, pairwise ~ week * treatment)

# Adjust for multiple testing, using Tukey method

summary(emm_interaction$contrasts, adjust = "tukey")


# Survival analysis-----------

survival <- read_excel("starvationdata.xlsx",

                 sheet = "survival")

View(survival)

attach(survival)


#Fit Kaplar Meier Model

km.model<-survfit(Surv(week, dead)~status)

summary(km.model)
```

```r
#Test for difference of groups

cox.model <- coxph(Surv(week, dead) ~ status, data = survival)

summary(cox.model)


#web density and pheromone analyses -------


webpher <- read_excel("starvationdata.xlsx",
                 sheet = "webpher")

View(webpher)

#format variable types

webpher$week<-as.numeric(webpher$week) #weeks of starvation

webpher$overall<-as.numeric(webpher$overall) #overall silk strand density per cubic cm

webpher$safety<-as.numeric(webpher$safety) #silk density in retreat corner per square cm

webpher$prey<-as.numeric(webpher$prey) #silk density of gum-footed lines per square cm

webpher$sp<-as.numeric((webpher$safety)/webpher$prey) #ratio of safety and prey capture silk investment

webpher$ps<-as.numeric((webpher$prey)/(webpher$safety)) #ratio of safety and prey capture silk investment

webpher$cPher<-as.numeric(round(webpher$cPher, digits = 0)) #contact pheromone components

webpher$Amide<-as.numeric(round(webpher$Amide, digits=0)) #serine-amide breakdown product as proxy for mate-call components

webpher$treatment<-as.factor(webpher$treatment) #treatment fed or starved

webpher$spider<-as.character(webpher$spiderID) #spider individual

webpher$tcPher<-as.numeric(round((webpher$cPher/273) + (webpher$Amide/203)*273, digits=0)) #total deposited pheromone titer = cPher + Amide in Stoffmenge and then back in mass

webpher$breakdown<-(webpher$Amide/203)/((webpher$cPher/273) + (webpher$Amide/203)) #Breakdown ratio of pheromone = mate call investment

webpher$normpher<-as.numeric(round((webpher$cPher/webpher$overall)), digits=0) #web density normalized contact pheromone titer

View(webpher)
```

```
attach(webpher)

#Model overall web density change by treatment over time.
webdensity <- glmmTMB(overall ~ week*treatment + (1|spider), data = webpher, family = tweedie())
#testing assumptions
obj <- simulateResiduals(webdensity,plot=F)
plot(obj, quantreg = F)
#testing for significance of terms
Anova(webdensity,type="III")
# Conduct pairwise comparisons between treatments at week 28
contrast(emmeans(webdensity, specs = pairwise ~ treatment | week, at = list(week = 28)), method = "pairwise", simple = "treatment")

#Model safety silk investment change by treatment over time.
safety <- glmmTMB(safety ~ week*treatment + (1|spider), data = webpher, family = tweedie())
#testing assumptions
obj <- simulateResiduals(safety,plot=F)
plot(obj, quantreg = F)
#testing for significance of terms
Anova(safety,type="III")

#Model preycapture silk investment change by treatment over time.
prey <- glmmTMB(prey ~ week*treatment + (1|spider), data = webpher, family = tweedie())
#testing assumptions
obj <- simulateResiduals(prey,plot=F)
plot(obj, quantreg = F)
#testing for significance of terms
Anova(prey,type="III")
```

```
#Model safety/preycapture investment ratio change by treatment over time.

ps <- glmmTMB(ps ~ week*treatment + (1|spider), data = webpher, family = tweedie())

#testing assumptions

obj <- simulateResiduals(sp,plot=F)

plot(obj, quantreg = F)

#testing for significance of terms

Anova(ps,type="III")


#Model contact pheromone titer change by treatment over time.

cPher <- glmmTMB(cPher ~ week*treatment + (1|spider), data = webpher, family = nbinom1())

#testing assumptions

obj <- simulateResiduals(cPher,plot=F)

plot(obj, quantreg = F)

#testing for significance of terms

Anova(cPher,type="III")


# Conduct pairwise comparisons between treatments at week 28

contrast(emmeans(cPher, specs = pairwise ~ treatment | week, at = list(week = 28)), method =
"pairwise", simple = "treatment")


#Model mate call release change by treatment over time

amide<- glmmTMB(Amide ~ week*treatment + (1|spider), data = webpher, family = nbinom2())

#testing assumptions

obj <- simulateResiduals(amide,plot=F)

plot(obj, quantreg = F)

#testing for significance of terms

Anova(amide,type="III")

# Conduct pairwise comparisons between treatments at week 28
```

```
contrast(emmeans(amide, specs = pairwise ~ treatment | week, at = list(week = 28)), method = "pairwise", simple = "treatment")


breakdown<- glmmTMB(breakdown ~ week*treatment + (1|spider), data = webpher, family = tweedie())

#testing assumptions

obj <- simulateResiduals(breakdown,plot=F)

plot(obj, quantreg = F)

#testing for significance of terms

Anova(breakdown,type="III")

# Conduct pairwise comparisons between treatments at week 28

contrast(emmeans(breakdown, specs = pairwise ~ treatment | week, at = list(week = 28)), method = "pairwise", simple = "treatment")



normpher<-glmmTMB(normpher ~ week*treatment + (1|spider), data = webpher, family = tweedie())

#testing assumptions

obj <- simulateResiduals(normpher,plot=F)

plot(obj, quantreg = F)

#testing for significance of terms

Anova(normpher,type="III")

# Conduct pairwise comparisons between treatments at week 28

contrast(emmeans(normpher, specs = pairwise ~ treatment | week, at = list(week = 28)), method = "pairwise", simple = "treatment")



#Y-tube and mating trials------

##Ytube choices (x, N, one/two sided)----

#fed vs control (one sided)

binom.test(13,18, alternative="greater") #test for attraction
```

```
binom.test(13,18, alternative="less") #control for repellent-effect
#p value fed vs. control
binom.test(13,18, alternative="greater")$p.value

#staved vs control (one sided)
binom.test(16,20, alternative="greater") #test for attraction
binom.test(16,20, alternative="less") #control for repellent-effect
#p value starved vs. control
binom.test(16,20, alternative="greater")$p.value

#fed vs starved (two sided)
binom.test(9,18)
binom.test(9,18)$p.value

## calculate body indices
# female body indices
mating <- read_excel("starvationdata.xlsx",
          sheet = "mating")
View(mating)
attach(mating)
#female body condition at 0W starvation
F0<-lm(fem0Mass ~ femSize)
summary(F0)
par(mfrow=c(2,2))
plot(F0)
plot(fem0Mass~femSize)
mating$fem0Cond0<-F0$residuals
View(mating)
#female body condition at mating time (~24W starvation)
```

```
FM<-lm(FemMatingMass ~ femSize)

summary(FM)

par(mfrow=c(2,2))

plot(FM)

plot(FemMatingMass~femSize)

mating$femMatingCond<-FM$residuals

View(mating)

#male body condition at mating time

male<-lm(maleMass ~ maleSize)

summary(male)

par(mfrow=c(2,2))

plot(male)

plot(maleMass ~ maleSize)

mating$maleCond<-male$residuals

View(mating)

##mating experiment variables-----

mating$Treatment<-as.character(mating$Treatment) #starved (S) or fed (F)

mating$Success<-as.numeric(mating$Success) # occurrence of copulation, "1"=copulation, "0" no copulation

mating$Cannibalism<-as.factor(mating$Cannibalism) # occurrence of cannibalism, "1"=cannibalism, "0" no cannibalism

mating$durCop<-as.numeric(mating$durCop) #duration of copulation in min

mating$durCourt<-as.numeric(mating$durCourt) #duration of male courtship in min

mating$matingAge<-as.numeric(mating$matingAge) #female age at time of mating

mating$offspring<-as.numeric(mating$offspring) #number of live offspring hatched during life of mother

attach(mating)


##ensure treatment pairs are appropriately chosen

wilcox.test((matingAge~ Treatment), exact=F)#test for female age difference across the pairs
```

```
wilcox.test((fem0Cond~ Treatment), exact=F)#test for female condition difference  across the pairs at time of pairing

wilcox.test((femMatingCond~ Treatment), exact=F)#test for female condition difference across the pairs at time of mating

wilcox.test((maleCond~ Treatment), exact=F)#test for male condition difference across the pairs


#data analysis

##Compare number of offspring produced by fed and starved females

wilcox.test((offspring~ Treatment), exact=F)


##compare matting occurrence (success) between fed and starved females

table(Treatment,Success)

fisher.test(Success, Treatment)


##compare sexual cannibalism occurrence

table(Treatment,Cannibalism)

fisher.test(Cannibalism, Treatment)


##compare duration of male courtship

wilcox.test((durCourt~ Treatment), exact=F)


#descriptives

summary <- mating %>%

 group_by(Treatment) %>%

 summarise(

  Mean = mean(durCourt, na.rm = TRUE),

  SE = sd(durCourt, na.rm = TRUE) / sqrt(n()),

  Median = median(durCourt, na.rm = TRUE)

 )
```

```
summary

##compare duration of copulation
shapiro.test(durCop)
t.test(durCop~ Treatment)
#descriptives
summary <- mating %>%
  group_by(Treatment) %>%
  summarise(
    Mean = mean(durCop, na.rm = TRUE),
    SE = sd(durCop, na.rm = TRUE) / sqrt(n()),
    Median = median(durCop, na.rm = TRUE)
  )
summary
```
